# Detection of Perturbation Phases and Developmental Stages in Organisms from DNA Microarray Time Series Data

**DOI:** 10.1371/journal.pone.0027948

**Published:** 2011-12-15

**Authors:** Marianne Rooman, Jaroslav Albert, Yves Dehouck, Alexandre Haye

**Affiliations:** 1 BioSystems, BioModeling and BioProcesses Department, Université Libre de Bruxelles, Bruxelles, Belgium; 2 Complex Systems Group, Fritz-Haber-Institut der Max-Planck Gesellschaft, Berlin, Germany; University of Sheffield, United Kingdom

## Abstract

Available DNA microarray time series that record gene expression along the developmental stages of multicellular eukaryotes, or in unicellular organisms subject to external perturbations such as stress and diauxie, are analyzed. By pairwise comparison of the gene expression profiles on the basis of a translation-invariant and scale-invariant distance measure corresponding to least-rectangle regression, it is shown that peaks in the average distance values are noticeable and are localized around specific time points. These points systematically coincide with the transition points between developmental phases or just follow the external perturbations. This approach can thus be used to identify automatically, from microarray time series alone, the presence of external perturbations or the succession of developmental stages in arbitrary cell systems. Moreover, our results show that there is a striking similarity between the gene expression responses to these *a priori* very different phenomena. In contrast, the cell cycle does not involve a perturbation-like phase, but rather continuous gene expression remodeling. Similar analyses were conducted using three other standard distance measures, showing that the one we introduced was superior. Based on these findings, we set up an adapted clustering method that uses this distance measure and classifies the genes on the basis of their expression profiles within each developmental stage or between perturbation phases.

## Introduction

In higher eukaryotes, the life span is separated into discrete developmental phases that start from the embryonic phase and end with the adult phase, and are in some organisms separated by other stages such as larval and pupal stages. On the other hand, the gene expression levels of an organism evolve with time and this time evolution can be inferred from appropriate DNA microarray time series. The question we ask here is: can we infer the limits of the developmental phases from the gene expression profiles alone, in other words, is there a sudden change in behavior that is discernable in the profiles?

Furthermore, both unicellular and multicellular organisms may be subject to external perturbations, which trigger a specific gene expression response. Abrupt temperature changes, oxidative stress or the addition of particular molecules are examples of such perturbations. A change in the amount of nutrients is another example. Bacteria for instance are usually able to grow on different (usually two) kinds of sugars, but need to exhaust their preferred sugar before using the others, in a phenomenon called diauxie. The second question we ask here is whether we can also infer solely from the gene expression profiles the exact time point where the cells are subject to such external perturbations. The corollary question is whether this response appears to be different than for successive developmental stages.

The possibility of detecting the limits of the developmental stages of higher eukaryotes from the gene expression profiles is analyzed here on the basis of model organisms for which long enough microarray time series are available, *i.e.* sea squirt, vinegar fly, silkworm and mouse. The detection of external perturbations is performed on several *E. coli* DNA time series subject to heat, cold and oxidative stress and to glucose-lactose diauxie. The approach is simple: the shapes of the gene expression profiles are compared over a few successive time points, and regions of large changes are identified as regions where developmental stage modifications or external perturbations occur.

This approach leads us to design an appropriate clustering procedure, which consists of dividing profiles into subprofiles at the time points where sudden changes in the expression levels occur, and to group genes in the same class when they have similar subprofiles.

## Methods

### 1. Gene expression profiles

#### 1.a Measured profiles

DNA microarray time series yield the concentrations of all or a subset of the RNAs that are present in a given cell sample at *N* different time points *t_i_* (*i* = *1*,..*N*). These RNAs, labeled by μ, may be mRNAs or miRNAs. Their concentrations are estimated by converting them into cRNAs or cDNAs, labeling these by fluorophores and measuring the fluorescence intensities 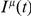 emitted when they are hybridized to their complementary sequence attached to a microarray. These intensities are often given relative to a reference intensity 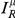, which depends on the RNA but not on the time, and is measured from an unperturbed sample or a mixture of several samples. As the measures come from different hybridizations, they must be normalized to correct for different effects including the unequal quantities of starting RNA, differences in labeling or detection efficiencies between the fluorescent dyes used, and systematic biases in the measured expression levels [1]–[2]. The gene expression profiles 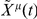 we consider here are defined as a function of the normalized intensities 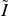 as:
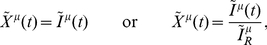
(1)depending on the available data. We made here the common assumption that the RNA concentrations and the normalized fluorescence intensities are proportional [3]. In what follows, the index *μ* will refer indistinguishably to the RNA or the gene from which it is transcribed.

#### 1.b Development of multicellular eukaryotes

DNA microarray time series that monitor the different developmental stages of multicellular eukaryotes and possess a sufficient number of time points per stage are available for the vinegar fly *Drosophila melanogaster*, the urochordate *Ciona intestinalis*, the silkworm *Bombyx mori* and the mouse *Mus musculus*.

The *Drosophila melanogaster* DNA microarray time series [4] yields the expression levels of 4,028 genes across all four developmental phases. Among the 67 time points, 31 are in the embryonic phase (covering 24 hours; the first 14 points are taken every half hour, and the last 17, every hour; the measuring period is equal to one hour, so that the former 14 measures overlap), 10 are in the larval phase (spanning 81 hours in approximately 9 hour intervals), 18 in the pupal phase (96 hours; 7 points every 2 hours, 3 points every 4 hours, 4 points every 6 hours, 2 points every 12 hours, one point after 8 hours, and one point after 16 hours), and 8 in the adult phase (30 days; 3 points every 2 days, 5 points every 5 days). Each of these 67 samples was compared with a unique reference sample, consisting of a standard mixture of all samples of the series. Only the time series for male flies was considered in this paper. However, we also tested the female flies' time series and obtained very similar results; the only differences lie in the adult phase. A subset of 20 genes has been shown to be related to muscle development [5] and has been analyzed separately.

The *Ciona intestinalis* DNA microarray time series [6] monitors the expression levels of 21,938 genes during the life cycle. It contains a total of 18 time points: 13 in the embryonic phase (17 hours), 1 in the larval phase, and 4 in the adult phase (4 months). All these expression levels were given relative to the same reference sample, corresponding to fertilization, except the latter four points, which were each given relative to the previous point. To obtain meaningful profiles from these time points, we chose the first (fertilization) point as a reference, and multiplied the expression levels at the four time points corresponding to the adult stage by the expression level of the previous point. We hence obtained a series of 18 time points with a unique reference sample.

Two oligonucleotide-based DNA microarray time series of the mouse *Mus musculus* were considered. The first [7] reveals the expression pattern of 6,579 genes throughout the morphologic stages of lung development. It consists of a total of 11 time points, 4 in the embryonic stage, 6 in the postnatal stage and 1 in the adult stage. The other time series [8] is focused on the mammary gland development. It monitors the expression of 12,488 genes over 18 time points, covering the virgin (3 points), pregnancy (7 points), lactation (3 points), and involution (5 points) stages. In the involution stage, the mammary gland undergoes complex processes of controlled apoptosis and tissue remodeling. The data used here corresponds to the average over 3 replicas.

The silkworm *Bombyx mori* undergoes four distinctive main developmental stages, defined as embryo, larva, pupa, and adult moth, which are monitored by a DNA microarray series of 41–42 time points [9]: 8 in the embryonic stage, 20 in the larval stage, 1 in the prepupal stage, 10 in the pupal stage and 2 or 3 in the adult stage. Two replicas are analyzed and their average is taken. Female and male worms are considered separately, from the end of the larval stage. In contrast to all other series considered in this paper, which measure mRNA concentrations, this series profiles miRNA expression. A total of 106 miRNAs are considered.

Note that in several of the above listed series the cell samples were taken indistinguishably from any part of the organism and thus represent an average of the gene expression levels in the different tissues. In these cases, the measurements thus mix the dependencies of the expression levels on the organism's developmental stage and on the cell's host tissue.

#### 1.c External perturbation of unicellular systems

DNA microarray time series that monitor the response of gene expression levels upon perturbations have been considered for *Escherichia coli*.

A first kind of external perturbation is glucose–lactose diauxie, which is monitored in *E. coli* through a whole-genome DNA array time series [10]. A total of 4,289 genes and 17 time points were considered, 3 before the diauxic lag, 10 during the growth on lactose and 4 after lactose exhaustion. There are thus two different phases of growth arrest, a transient one after depletion of glucose, during the diauxic lag, and another after depletion of lactose.

Other kinds of environmental fluctuations, in particular cold, heat and oxidative stress, were studied by DNA microarray time series in *Escherichia coli* monitoring the expression profiles of 4,400 genes [11]. A total of 12 time points was considered for oxidative stress and 8 time points for cold and heat stress, covering the periods before stress, during growth arrest due to the stress, and during growth resumption. The last period corresponding to the stationary phase was considered after oxidative stress. For each of these perturbations 3 replicas were considered and their average was taken.

#### 1.d Cell cycle

The gene expression levels along the cell cycle have been monitored in the yeast *Saccharomyces cerevisiae* by three DNA microarray time series, in which the cells were synchronized by three independent methods: a factor arrest, elutriation, and arrest of a *cdc15* temperature-sensitive mutant [12]. These series cover two to three successive cell cycles (16 time points for elutriation, 18 time points for a factor arrest, and 25 time points for *cdc15*), and profile more than 6,000 genes.

### 2. Detection of perturbation points in expression profiles

The hypothesis we test here is that the limits of the developmental stages of higher eukaryotes appear in the gene expression profiles as regions where the expression levels undergo some kind of change. Similarly, the expression levels are also expected to undergo modifications in response to stress or other external perturbations. The kind of change that is expected to occur in such particular regions is not obvious *a priori*. Expression levels generally vary over time (except in stationary phases), often even in the absence of perturbations of any kind. We therefore do not search for changes in the expression levels of each gene individually. Rather, we choose to compare the profiles of the different genes, and detect time intervals where the variety of profiles is larger than on the average. Such a phenomenon could indeed be indicative of an uncoordinated response of the expression patterns to some general perturbation.

To detect such a response, an appropriate distance measure between segments of gene profiles must be defined. An important point is that this measure must be insensitive to the sampling frequency of time points. Indeed, this frequency depends on the experimental setup and is generally different according to the developmental stages. Its effect must thus be overlooked. We test here four different distances to measure the similarity between gene profiles, which are all independent of the sampling frequency. They are described below.

#### 2.a Euclidean distance

The first and simplest measure we consider is the Euclidean distance *D^E^* between two regions 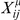 and 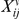 of the expression profiles 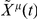 and 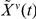 of genes μ and ν, which are contained between the time points *t_i_* and *t_j_* (*i<j*). It is given by:
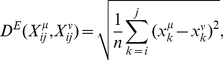
(2)where the profiles 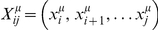 are taken at the *n*≡*j-i*+1 successive time points *t_k_*. This distance is symmetric, *i.e.* 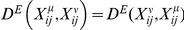, but is in general dependent on shifts and scaling factors, *i.e.* 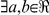: 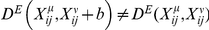 and 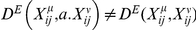.

#### 2.b Least-rectangle distance

Two profiles that are translated with respect to each other and thus present the same shape but different average expression levels can be assumed to be identical. Indeed, we search for true modifications in expression patterns rather than simple relative shifts. Furthermore, two profiles that are scaled with respect to each other can also be assumed to be similar. This is justified by the fact that expression levels are generally defined relative to a gene-dependent but time-independent reference expression level 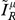, as shown in eq. (1). The scaling factor between two profiles may thus simply be due to the different reference expression levels, and lacks intrinsic meaning.

We thus define a new distance, noted *D^R^*, as the Euclidean distance between the profile region 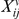 of gene *ν* and the equivalent region for gene *μ*, 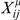, translated by 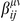 and scaled by 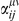. However, this distance is not symmetric. To obtain a symmetric expression, satisfying 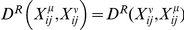, this new distance is defined as:
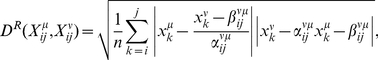
(3)with 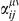,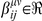. Negative values of 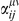 correspond to reflections 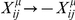. The values of these parameters are obtained by requiring that 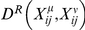 is minimum with respect to them, *i.e.* 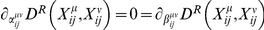. They are given as a function of the mean and standard deviation:
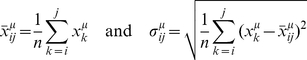
(4)as:
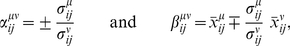
(5)where the sign that minimizes the distance is chosen. Inserting these values in eq. (3) yields:
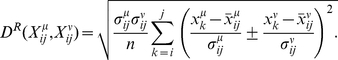
(6)This distance has the following geometrical interpretation. Consider the *n* points of coordinates 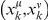 (*i≤k≤j, n = j-i+1*) in a plane with Cartesian coordinate system 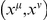. The equation 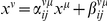 corresponds to the least-rectangle regression line for these points, which minimizes the deviations of both coordinates 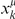 and 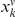 to the regression line. It is thus a symmetrized version of the least-square regression line, which only minimizes the deviation of 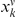 coordinates. We therefore call the distance defined by eq. (6) the least-rectangle distance. It is insensitive to translations and reflections, *i.e.* 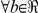: 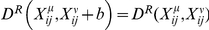 and 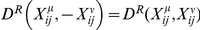. It is moreover scale-invariant with scaling dimension 1/2, *i.e.* 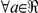
_:_ 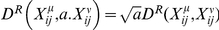. Note that, due to translation and scaling which define the superposition of the profiles, the least-rectangle distance values are always smaller than (or equal to) the corresponding Euclidean distance values.

A C-function that allows the computation of this distance within the software environment R [13], as well as example programs to compute the distance between profiles or the average distance between profile segments, can be downloaded at the address: http://babylone.ulb.ac.be/pubs/suppmat/least-rectangle-distance/.

#### 2.c Scaling-insensitive variant of the least-rectangle distance

A distance that is scale-invariant with scaling dimension zero, and is thus insensitive to the value of the scaling factor, *i.e.* 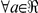
_:_ 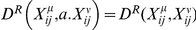, can easily be obtained from the least-rectangle distance. It reads as:
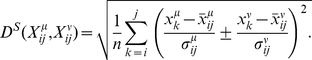
(7)This distance is symmetric, translation-, scale- and reflection-invariant, and insensitive to the value of the scaling factor.

#### 2.d Pearson correlation distance

An often-used distance to measure the similarity in shape between two profiles is the Pearson correlation distance, defined as:
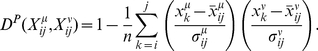
(8)This distance is symmetric and translation-invariant. It is scale-invariant for positive scaling factors, with scaling dimension equal to zero, *i.e.* 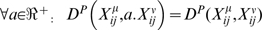. It is not invariant with respect to negative scaling factors, and thus under reflections.

#### 2.e Mean distance between profile regions

To detect if some sudden changes occur in the expression profiles of *M* genes, we compute the pairwise distance between the expression profiles of any two genes μ and ν, contained between the time points *t_i_* and *t_j_*, for fixed values of *n≡j-i+1*, using one of the four distances *D^E^*, *D^R^*, *D^S^* and *D^P^* defined above. We then compute the average over all genes. This yields:
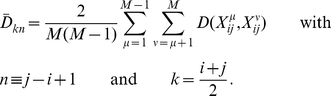
(9)We consider here distances between expression profile regions counting between 3 and 6 time points, *i.e. n* = 3,…6. Time points *t_k_* where 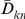 undergoes a sudden change indicate perturbation points.

## Results

### 1. Detection of developmental and perturbation phases in gene expression profiles

#### 1.a Selection of the distance measure

In view of investigating whether the limits of the developmental stages in higher eukaryotes and of the perturbation phases in cellular systems can be detected from the gene expression profiles, the mean distance between profile regions 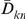 defined in eq. (9), with the four distance measures *D^E^*, *D^R^*, *D^S^* and *D^P^* given in eqs (2,6,7,8), is computed for all DNA microarray time series considered here (described in sections 1.b–d of Methods).

**Figure 1:**
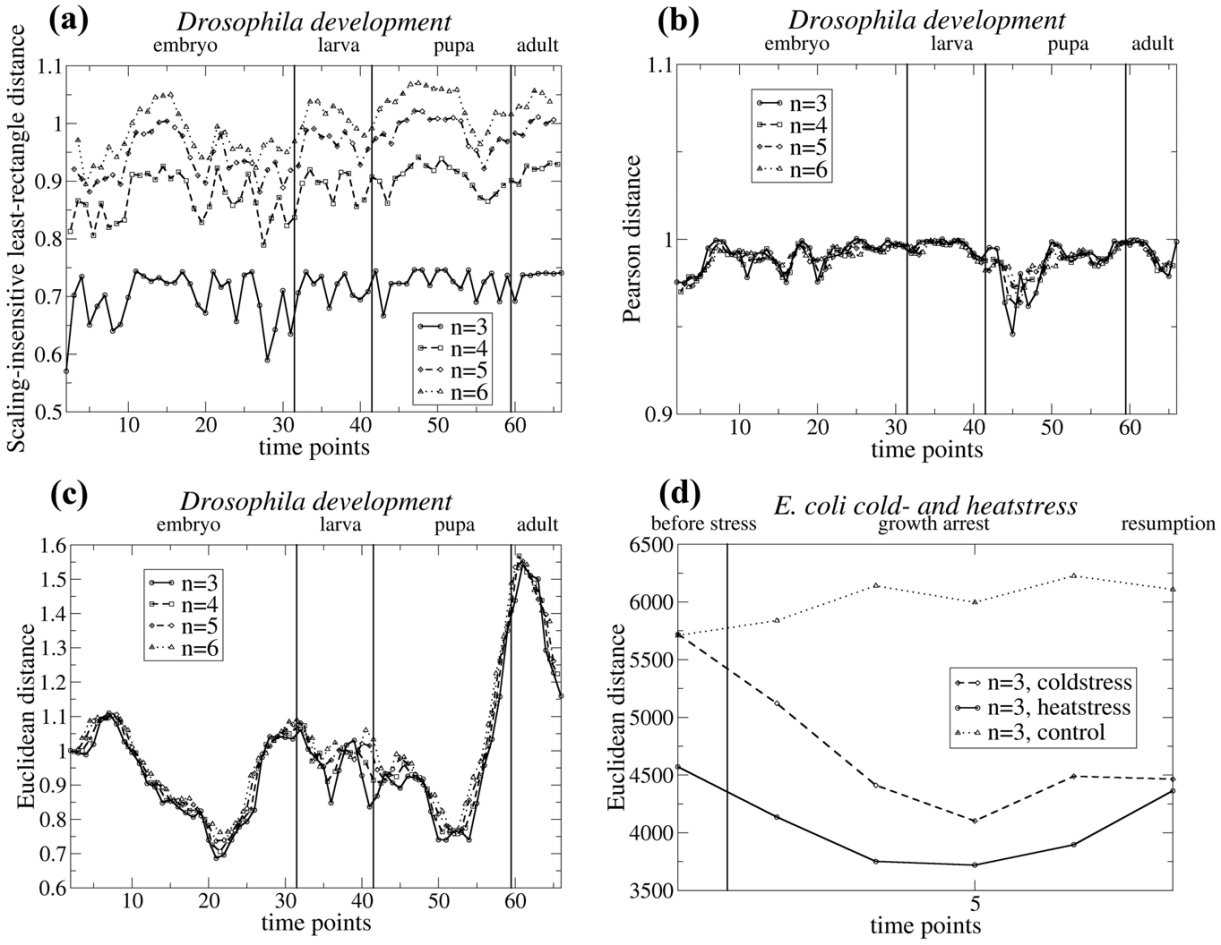
Average distance 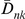 between profile segments of length *n*, as a function of the time points *k*, for gene expression profiles obtained from DNA microarray experiments. The values of *n* are indicated on the figures, as well as the developmental phases. Note that the values of the distances appear sometimes very different in the different graphs; this is due to the fact that some gene expression profiles 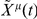 borrowed from the literature are scaled and/or expressed relative to a reference sample and others not (see eq. (1)). Different distance measure are used to compute 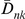: (a) scaling-insensitive variant of the least-rectangle distance *D^S^*; (b) Pearson distance *D^P^*; (c, d) Euclidean distance *D^E^*. These distances are computed on the DNA microarray time series monitoring: (a–c) *Drosophila* development; (d) unstressed *E. coli* (used as a control).

**Figure 2:**
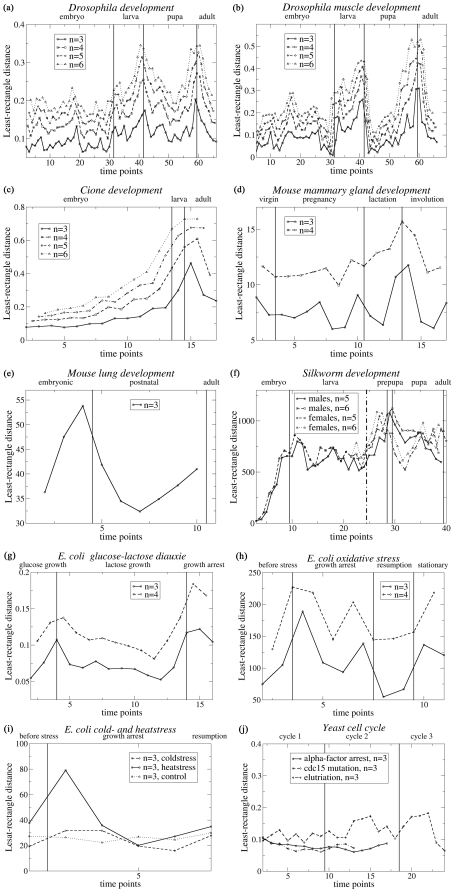
Average distance 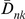 between profile segments of length *n*, as a function of the time points *k*, for gene expression profiles obtained from DNA microarray experiments. These average distances are computed with the least-rectangle distance measure *D^R^*. The values of *n* are indicated on the figures, as well as the developmental phases and perturbation points. Note that the values of the distances appear sometimes very different in the different graphs; this is due to the fact that some gene expression profiles 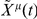 borrowed from the literature are scaled and/or expressed relative to a reference sample and others not (see eq. (1)). (a) *Drosophila* development; (b) *Drosophila* muscle development; (c) *Cione* development; (d) Mouse mammary gland development; (e) Mouse lung development; (f) Silkworm development; the vertical dotted line indicates the time point were males and females start to be distinguished; (g) *E. coli* subject to glucose-lactose diauxie; (h) *E. coli* subject to oxidative stress; (i) *E. coli* subject to cold- and heatstress, and to no stress; (j) *S. cerevisae* cell cycles.

The comparison of the four distance measures gives very similar results for all time series. Therefore, only the results for the series monitoring the *Drosophila* development are shown for all distances (Fig. 1a–c and Fig. 2a). When the average segment distance 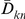 is computed with the Pearson correlation distance *D^P^* or with the scale-insensitive variant of the least-rectangle distance *D^S^*, no peaks are observed at all (Figs. 1a–b). Rather, 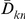 is almost constant with some noise-like fluctuations. These two distances are thus to be rejected, as they are unable to detect any transitions between different phases.

In contrast, the Euclidean distance *D^E^* and the least-rectangle distance *D^R^* present very clear peaks (Figs. 1c and 2a). In the case of the Euclidean distance, one peak appears between the pupal and adult stages, and another between the embryonic and larval stages. However, no peak appears at the transition between larval and pupal stages, and a clear peak appears some time after the beginning of the embryonic stage. This distance thus yields mixed results. In the other time series considered, the results obtained with the Euclidean distance are mixed too: peaks appear often but not always where expected. In particular, Fig. 1d shows the results on unstressed *E. coli*, where no peaks are expected to occur, as well as on *E. coli* subject to heat and cold stress, where peaks are expected after the stress. Contrary to the expectations, there are no peaks in the region of the perturbation; the highest values of the mean segment distance 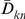 appear much before the stress. Moreover, for unstressed *E. coli*, which may be considered as a control series, no peaks appear but a constant increase is visible, which seems meaningless. The Euclidean distance *D^E^* is thus more informative than the Pearson correlation distance *D^P^* and the scale-insensitive variant of the least-rectangle distance *D^S^*, but is far from perfect and will not be further considered.

In the case of the least-rectangle distance *D^R^*, much clearer peaks appear at each transition between perturbation phases or development stages, for all time series, as shown in Fig. 2 and discussed in detail in the next section. Moreover no peaks and no increase are visible in the absence of such transitions, in particular in the control series describing unstressed *E. coli* (Fig. 2i). This distance is thus adequate to detect transitions from gene expression data, and will be analyzed further.

#### 1.b Development of multicellular eukaryotes

For the *Drosophila* development, monitored by the expression levels of either 4,028 genes (Fig. 2a) or a subset of 20 genes related to muscles (Fig. 2b), the results are very clear: peaks in the average segment distance 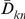 appear between the embryonic to larval, the larval to pupal and the pupal to adult stages. These peaks appear for all values of profile segment length *n* from 3 to 6. The last two peaks are particularly prominent. Smaller peaks appear within the embryonic stage, where the organism is known to change a lot and to pass through several substages. The largest peak of the embryonic stage is localized near time points 17–18, corresponding to about 12 hours after fertilization, at the end of the dorsal closure and the beginning of head involution [14]. This peak is even larger for the muscle profiles. This can be linked to the fact that 12 hours corresponds to the end of an important substage for muscle development, that is, the end of the development of muscle fibers presenting already the characteristics of the mature larval muscles [15].

For the *Cione* (Fig. 2c), one large peak in the average segment distance 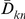 is visible, which encompasses the single time point in the larval stage, and is maximum at the very beginning of the adult stage. Here too, this distance measure allows the detection of the developmental phases. Note that, if several time points were available in the larval stage, this phase would probably be detected independently of the adult phase.

In the case of the mouse mammary gland development (Fig. 2d), a large peak is observed between the lactation and involution stages. In the latter stage, the mammary gland is known to undergo complex processes of controlled apoptosis and tissue remodeling. It is thus this important and sudden modification in the expression levels that our method detects. A small peak is also observed between the pregnancy and lactation stages for the profile segment length *n* = 3, but is much less significant. This indicates that the expression levels in the mouse mammary gland do not undergo sudden changes when passing from the virgin to pregnancy stage, undergo a small change from the pregnancy to lactation stage, and a very large change from the lactation to involution phase.

A very large peak in the average segment distance 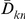 is observed at the very end of the embryonic stage, just before the postnatal stage, in the lung development in mouse (Fig. 2e). Moreover, a second peak starts at the end of the postnatal stage, but is not complete as there is only one time point in the adult stage. The limits of the known lung developmental stages are thus well detected by our method.

The last time series monitoring development is that of the silkworm (Fig. 2f). The first peak in 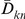 is observed between the embryonic and larval stages, and the second between the larval and prepupal/pupal stages. Not enough time points are available in the adult stage to see what happens at the beginning of this stage. Note that the peak between (pre)pupal and adult stages occurs earlier for the female than for the male silkworms. Other experiments are needed to determine whether this is a general phenomenon.

#### 1.c External perturbation of unicellular systems

In the *E. coli* glucose-lactose diauxie time series (Fig. 2g), two clear peaks in the average segment distance 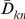 are observed: one after the exhaustion of glucose, and one after the exhaustion of lactose. The change in transcription network caused by these nutrient changes is very similar to that observed between developmental stages, when monitored by 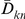.

Similar behaviors are observed for *E. coli* subject to stress. In the case of oxidative stress (Fig. 2h), a peak in the average segment distance 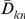 appears just after the stress, a second peak is observed just before the growth resumption, and a third one at the beginning of the stationary phase.

For heatstress (Fig. 2i), there is a very clear peak in 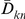 after the stress. The value of the distance increases again at the end of the time series, where growth starts to be recovered. For coldstress, the peak is much less pronounced, indicating that the transcription network is less modified. This means that passing from 37°C to 45°C requires more rewiring of the network than passing from 37°C to 16°C for *E. coli*. Note that the behavior of unstressed *E. coli* is also depicted in Fig. 1i as a control, and that in this case the average segment distance 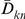 remains practically constant, as expected.

#### 1.d Cell cycle

The question was addressed whether sudden changes in expression levels occur within or at the end of cell cycles. To answer this, a time series covering several successive cycles in yeast was analyzed. As shown in Fig. 2j, no brusque changes in the average segment distance 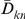 are observed. The distance values remain constant with small fluctuations that start to grow during the second and third cell cycles, where the cells start to be less well synchronized. So, no abrupt transcription network rewiring occurs during or after each cell cycle. Rather, continuous network remodeling is observed, where the expression level of different genes change at different times in the cycle, in agreement with earlier findings [16].

### 2. Stage-dependent clustering method

The gene expression profiles undergo sudden changes near the transition points between developmental stages and upon external perturbation. We are thus led to divide the profiles into subprofiles, each subprofile corresponding to a separate development or perturbation phase defined by the peaks in the 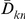 distances, and to cluster these subprofiles separately. The clustering is performed on the basis of the least-rectangle distance *D^R^* between subprofiles, defined in eq. (6). The clustering algorithm used is a simple hierarchical, tree-like, algorithm. It starts by considering each gene as a class on its own. It then joins, at each step, the two classes for which the average distance *D^R^* between any pairs of subprofiles from the two classes is minimum. It stops when all genes are in the same class. This clustering tree is then cut at a certain level by putting a threshold on the maximum number of classes, or on the average distance *D^R^* in the newly created class, so as to obtain an optimal number of clusters. The threshold has to ensure that the distances between subprofiles within each cluster are sufficiently low, and that the distances between subprofiles of different clusters are sufficiently high.

**Figure 3:**
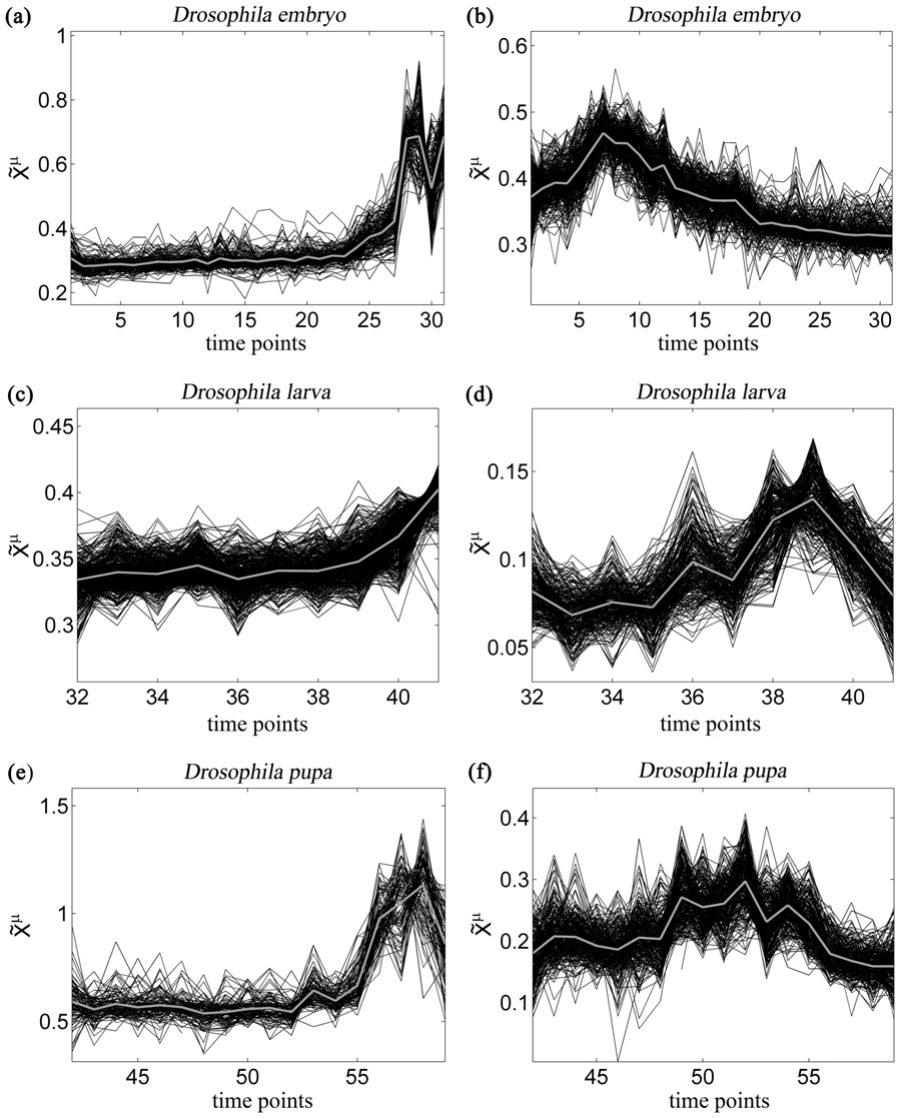
Examples of gene expression profiles 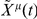 belonging to the same cluster, after suitable translation and dilatation to minimize the distance *D^R^*. The average profiles are indicated in gray. (a) Cluster of the embryonic stage, with 150 elements and <*D_intra_*> = 0.41; (b) Cluster of the embryonic stage, with 294 elements and <*D_intra_*> = 0.40; (c) Cluster of the larval stage, with 473 elements and <*D_intra_*> = 0.26; (d) Cluster of the larval stage, with 271 elements and <D_intra_> = 0.26; (e) Cluster of the pupal stage, with 97 elements and <*D_intra_*> = 0.40; (f) Cluster of the pupal stage, with 256 elements and <*D_intra_*> = 0.37.

To illustrate this method, we apply it to the embryonic, larval and pupal stages of *Drosophila*. We chose the number of classes within each stage to be equal to 10. Two of the clusters for each of these three stages are depicted in Fig. 3. As can be seen on these figures, the profiles in the same cluster exhibit a relatively small deviation from the average curve, owing to the translation- and scale-invariance of the distance *D^R^*. This is also visible from the values of the average distances between subprofiles within each cluster, <*D^R^*>_intra_, given in Table 1. Moreover, the average distance values between subprofiles of different clusters, noted as <*D^R^*>_inter_, are significantly higher than the corresponding <*D^R^*>_intra_, which indicates the reliability of the clustering. Note that the smaller values for the larval stage are due to the fact that the profiles in this stage exhibit much smaller variations.

**Table 1:** Average distances *D^R^* (in Å) between subprofiles within each cluster (<*D^R^*>_intra_) and between subprofiles of different clusters (<*D^R^*>_inter_) for the embryonic, larval and pupal stages of *Drosophila*.

Developmental stage	<*D^R^*>_intra_	<*D^R^*>_inter_
Embryo	0.41	0.56
Larva	0.25	0.33
Pupa	0.39	0.48

This classification leads us to describe the expression profile of each gene as a succession of representative subprofiles, one for each development stage or period between successive perturbation phases. Each representative subprofile represents a given cluster and corresponds to the average of all members of the cluster (see Fig. 3). This allows the description of the gene expression profiles that can sometimes be very complex as a series of quite simple curves, while keeping the trace of all the specificities and relevant peculiarities occurring in each stage. This classification also leads to the possibility of modeling the time evolution of gene expression separately in each stage, by considering different clusters in the different stages and connecting them at the perturbation points or between development phases.

## Discussion

The least-rectangle distance between expression profile segments, which is given by eqs (3–6) and is translation-invariant and scale-invariant with scaling dimension 1/2, appears to be a relevant measure for detecting perturbation or developmental phases from expression profiles. It allows the identification, on the basis of raw expression data alone, of time points where important phenomena take place, which lead to drastic rewiring of the gene expression network. Note that these expression data may involve all the genes in a system or a relevant subset, correspond to mRNA or miRNA, and come from cells of a specific tissue or a mixture of different cell types.

Other distance measures have been tested, but turned out to be unable to detect developmental and perturbation stages. In particular, the Pearson distance and a variant of the least-rectangle distance with scaling dimension zero yielded basically constant values of the average segment distance 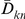, without any visible peaks. The Euclidean distance yielded 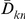 profiles with some clear peaks, but not always at the right position. It clearly contains some information, but is not the most adequate distance to detect transitions between perturbation phases or developmental stages. Only the least-rectangle distance gave the desired results. This distance is translation-invariant and scale-invariant, whereas the Euclidean distance is not. The other two distances exhibit both invariances but with scaling dimension zero, which makes them insensitive to the value of the scaling factor. This suggests that the translation invariance of the least-rectangle distance as well as its scale invariance with a scaling dimension of 1/2, are the relevant properties that allow the correct detection of transition points.

We tested yet other approaches, but without success. These include the estimation of parameters that measure the changes in each gene profile separately, such as the maximum difference in expression level at neighboring time points, and the sum of the Euclidean distance between the expression levels at successive time points in an interval [t_i_, t_j_] divided by the distance between the expression levels at t_i_ and t_j_. Note that measures that even implicitly depend on the sampling frequency can sometimes appear to be very efficient for detecting the limits of development stages. However, they have to be rejected, as they just demonstrate that the sampling frequency is often different in the different development stages. Another approach that we have tested consists of approximating the profiles by *P* polynomials of a fixed degree *d*, where *P* is equal to the number of stages and *d* is chosen between 1 and 5. Requiring that they do not overlap, cover the complete profile and present a minimal deviation from the profile identifies the optimal connection points between the *P* polynomials. These points are then compared with the changes in development stages or with the perturbation points. All these methods sometimes give positive results for certain systems and for certain stages, but never systematically.

Our method, based on the average segment distance 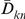 computed with the least-rectangle distance *D^R^*, appears thus as the only one suited to identify automatically, without prior knowledge, the time points where abrupt transcription network rewiring occurs, which corresponds to the passage to the next developmental stage or to a strong external perturbation. Note that for our method to be applicable, a sufficient number of time points must be available for each phase. Note also that the optimal length *n* of the profile segments that allows the best detection of the transitions between the phases depends on the number of time points. When many time points are available for each phase, the peaks appear more clearly for values of *n* equal to 5 or 6, as seen in Figure 2. In contrast, for phases with few time points, the peaks are averaged out and disappear for *n = *5–6. In such cases, *n*-values of 3 or 4 must be considered.

An interesting result is that we cannot distinguish, from the DNA microarray expression data, the response to an external perturbation from the succession of developmental stages. The only difference that can be noted is that the distance peak follows the perturbation whereas it usually occurs at the same time as, or slightly before, the changes in developmental stage.

These results are consistent with the idea that some (unknown) external or internal perturbation affects the gene expression network at the end of each developmental stage, and triggers it towards the next stage. It has been argued that a cell system approaches a fixed point, a limit cycle or another type of attractor at specific moments of its life [16]–[17]. This could be the case at the end of each developmental stage. In the adult stage, the approach to an attractor certainly appears as a reasonable hypothesis, since the expression profiles become almost stationary in the absence of external stimuli. The careful observation of the expression profiles leads us to think that this might also be true at the end of each developmental phase, but more precise data should be available to deepen this hypothesis. If this vision were true, it would provide a fair and simple explanation of the extreme robustness of cell systems with respect to stochastic perturbations of all kinds. Only when the system is perturbed in a specific way does it evolve to another attractor. Note, however, that a system is never totally robust. It can always undergo specific perturbations that lead it towards unwanted attractors, which could for example be the origin of cancer-like diseases.
